# Influence of Sex Differences on Serum Lipid Profiles among Habitual Coffee Drinkers: Evidence from 23,072 Taiwan Biobank Participants

**DOI:** 10.3390/nu15112576

**Published:** 2023-05-31

**Authors:** Meng-Ying Lu, Jerry Cheng-Yen Lai, Shaw-Ji Chen

**Affiliations:** 1 Division of Cardiology, Department of Internal Medicine, Taitung MacKay Memorial Hospital, Taitung 95054, Taiwan; davielu.tw@yahoo.com.com; 2 Department of Medicine, MacKay Medical College, New Taipei City 25245, Taiwan; 3 Master Program in Biomedicine, College of Science and Engineering, National Taitung University, Taitung 95092, Taiwan; chengyen@hotmail.com; 4 Department of Medical Research, Taitung MacKay Memorial Hospital, Taitung 95054, Taiwan; 5 Department of Psychiatry, Taitung MacKay Memorial Hospital, Taitung 95054, Taiwan

**Keywords:** coffee, sex, lipid profile, menopause

## Abstract

The bioactive compounds of coffee are involved in lipid metabolism, and sex differences may play an important role. This study aimed to evaluate the influence of sex differences on serum lipid profiles among habitual coffee drinkers. We conducted a nationwide cross-sectional study of 23,628 adults using data obtained from the Taiwan Biobank database. Adults who drank more than one cup of coffee per day and those who drank less than one cup per day were compared with non-drinkers. After adjusting for baseline demographics and lifestyle, a generalized linear model was used to estimate the change in serum lipid profiles in men and women and in postmenopausal and premenopausal women among different coffee-drinking behaviors. We found that habitual coffee consumption changed the serum lipid profiles of men and women. Further, coffee drinkers had higher serum total cholesterol, low-density lipoprotein cholesterol, high-density lipoprotein cholesterol, and lower serum triglyceride levels than non-drinkers. Compared with premenopausal women, both men and postmenopausal women had increased serum total cholesterol and low-density lipoprotein cholesterol levels. Menopausal status may play an important role in modulating the effect of habitual coffee intake on dyslipidemia. Moreover, premenopausal women potentially benefit more from habitual coffee drinking than men and postmenopausal women.

## 1. Introduction

Coffee is a popular worldwide beverage containing hundreds of bioactive compounds, including caffeine, chlorogenic acid, lignans, the alkaloid trigonelline, and diterpenes [1]. Moreover, it may bring habitual drinkers potential health benefits or risks [2]. Many studies have discussed the effects of diterpenes, including cafestol and kahweol, on the serum cholesterol levels of habitual coffee drinkers [3,4]. Although diterpenes have been regarded as cholesterol-raising compounds, the serum cholesterol levels of habitual coffee drinkers do not always significantly increase, since there are many factors, such as brewing methods, coffee bean varieties, and the volume of daily consumption, that affect the influence of diterpenes on serum cholesterol [1,2,5,6]. Conversely, low-density lipoprotein cholesterol (LDL-C) is regarded as a major risk factor for atherosclerotic cardiovascular disease [7]. Further, the potential risk of cholesterol increase from habitual coffee drinking may have important public health implications due to its increasing consumption in Taiwan. In previous studies, serum total cholesterol (TC) and LDL-C levels were found to be higher in habitual coffee drinkers who consumed unfiltered coffee than in those who consumed filtered coffee [8,9,10], possibly due to the diterpenes being filtered out through the filter paper. However, the concentration of diterpenes varies among the different brewing methods and coffee types. For example, boiled Scandinavian and Turkish coffees contain much higher concentrations of diterpenes than filtered coffee, while espresso coffee contains intermediate amounts of diterpenes [11,12]. Arabica coffee contains higher levels of diterpenes than Robusta coffee [2]. Most studies on the effect of coffee on serum LDL-C levels have been conducted in Europe and America [4]; however, East Asians, such as the Taiwanese, have more diverse coffee drinking habits, especially habitual intake volume and coffee preference [13].

Studies have shown that lipid metabolism may be modulated by hormones and genetic variants due to gender [14,15,16,17]. Moreover, a potential risk of dyslipidemia in postmenopausal women with excess coffee drinking has been suspected [18]. Our study aimed to evaluate the effect of habitual coffee consumption on serum lipid profiles in adult Taiwanese men and women (including premenopausal and postmenopausal women) using the Taiwan Biobank (TWB) database.

## 2. Materials and Methods

### 2.1. Study Population

The TWB was established to collect lifestyle and health information from the Taiwanese population, and was sponsored by the Taiwanese government [19,20]. The population-based dataset was designed to recruit 200,000 healthy community-based participants aged 30–79 years. In 2020, data from 149,280 participants who voluntarily shared their data with the TWB were collected from 29 recruitment centers. In addition to blood sampling and physical examination, each participant completed a structured questionnaire on personal information and lifestyle factors during face-to-face interviews with a TWB researcher. For the current study, data were collected on 23,628 Taiwanese adults (8208 men and 15,420 women) aged 30–79 years without a diagnosis of dyslipidemia or current lipid-lowering drug use. All participants in the TWB provided written informed consent before data collection. Ethical approval for this study was obtained from the Institutional Review Board (IRB) of Mackay Memorial Hospital, Taipei, Taiwan (21MMHIS351e).

### 2.2. Habitual Coffee Drinking

The habitual coffee drinking questionnaire included the daily volume of coffee consumption. If the participants drank coffee three or more times per week, they were defined as coffee drinkers [21]. According to the mean daily intake volume (where one cup of coffee = 236.59 mL (8 fluid ounces)) [22], the coffee-drinking behavior of the participants was classified as follows: no habitual coffee drinking, less than one cup of coffee per day (<1 cup/day), and at least one cup of coffee per day (≥1 cup/day). 

### 2.3. Covariates

The information regarding age, sex, marital status, education level, occupational status, monthly income, residential area, alcohol consumption, habitual tea consumption, regular exercise, daily sleep duration (hours), smoking status, use of exogenous hormone supplements, and menopausal status were collected via questionnaires. Total cholesterol, high-density lipoprotein cholesterol (HDL-C), LDL-C, and triglyceride (TG) levels were determined using biochemical examinations. 

Body weight and height were obtained through physical examination, and body mass index (BMI) was calculated as weight (kg) divided by height squared (m^2^). The BMI was divided into three categories, including underweight–normal, overweight, and obese (BMI < 24.0, 24.0–26.9, and >27.0 kg/m^2^, respectively) [23]. 

Education level was categorized as graduation from college or higher or senior high school education. Marital status was categorized as either married or other (i.e., single, divorced, separated, or widowed). Occupational status categories included employed and unemployed individuals. Residential area categories included urban, suburban, and rural areas [24]. The monthly income level categories were classified as TWD 0–30,000, 30,000–60,000, and ≥60,000 per month [25]. The mean daily sleep duration included weekdays and weekends [26]. The participants who maintained a daily vegetarian diet (abstaining from the consumption of red meat, poultry, seafood, insects, and the flesh of any other animal) for at least six months before data collection were defined as vegetarians [27]. The enrollers who drank tea at least once a day were defined as habitual tea consumers [21].

Those who exercised for more than 150 min per week were regarded as the regular exercise group [28,29]. Non-smokers were defined as those who had never smoked or had not continuously smoked for ≥6 months. At the time of data collection, those who had smoked for at least six months consistently and were currently smoking or had stopped smoking were referred to as current and former smokers, respectively [30]. The alcohol consumption categories included non-drinkers, former drinkers, and current drinkers (0–150 mL/week alcohol intake for 6 months, no alcohol intake for >6 months, and ≥150 mL/week alcohol intake for six consecutive months) [21]. Postmenopausal status was determined retrospectively after the women had experienced 12 months of amenorrhea [31]. Exogenous hormones included oral contraceptives or hormone replacement therapy [32], and anyone with regular use of exogenous hormones for at least 6 months was recognized as a user in our questionnaire. 

### 2.4. Statistical Analysis

The characteristics of participants with different coffee-drinking behaviors were described as means and standard deviations for continuous data and as frequencies with percentages for categorical data. Each continuous variable was verified for normality of distribution using the Shapiro–Wilk test. All variables in the different groups were compared using one-way analysis of variance (ANOVA) for continuous data and chi-square tests for categorical data. Post hoc analysis was conducted for continuous variables in each group. After adjusting for baseline demographics and lifestyle, a generalized linear model (GLM) was used to estimate the change in serum lipid profiles in men and women with different coffee-drinking behaviors. Moreover, a GLM was applied to compare women with and without menstrual periods. Participant characteristics with *p* < 0.05 constituted the independent variables that were adjusted for in the generalized linear model. All data transformations and statistical analyses were performed using IBM SPSS for Windows (version 25; Armonk, NY, USA). The null hypothesis was rejected at an alpha level of 0.05.

## 3. Results

The general characteristics of the TWB participants, stratified by sex and daily coffee consumption volume, are shown in Table 1. The number of participants consuming coffee volumes of 0 cups/day, <1 cup/day, and >1 cup/day was 4887 (59.5%), 1558 (19.0%), and 1763 (21.5%) among men and 8971 (58.2%), 3003 (19.5%), and 3446 (22.3%) among women, respectively. The mean age of the participants who consumed coffee volumes of 0 cups/day, <1 cup/day, and >1 cup/day were 55.13 ± 11.04, 54.65 ± 10.79, and 53.12 ± 10.71 years among men, and 54.81 ± 10.18, 54.34 ± 9.72, and 51.80 ± 9.45 years among women, respectively. Both men and women had significant differences in lipid profiles, including TC, LDL-C, HDL-C, and TG, among the three groups with diverse coffee-drinking behaviors, except for TG in male participants. Moreover, there was a significant difference between age and BMI among men and women. Most of the personal baseline characteristics showed significant differences among the groups with different daily coffee consumption, except for marital status and place of residence in women and vegetarianism and daily sleeping hours in men.

The adjusted serum lipid profile changes based on coffee consumption are shown in Table 2. For the male group, compared with non-drinkers, all of the lipid profiles had significant changes in the group that consumed ≥1 cup/day (TC 4.52 mg/dL (95% confidence interval (CI) [2.63–6.40]); LDL-C 5.10 mg/dL (95% CI [3.42–6.79]); HDL-C 0.95 mg/dL (95% CI [0.37–1.53]); TG 9.62 mg/dL (95% CI [4.17–15.07])). However, in the group that consumed <1 cup/day, only TC and LDL-C had significant changes (TC 2.41 mg/dL (95% CI [0.45–4.38]); LDL-C 2.75 mg/dL (95% CI [0.99–4.51])). On the other hand, for the female group, all lipid profiles had significant changes in the group with habitual coffee consumption, either drinking <1 cup/day or ≥1 cup/day. TC significantly increased (3.53 mg/dL (95% CI [2.14–4.93]) and 4.83 g/dL (95% CI [3.49–6.17])), LDL-C significantly increased (2.88 mg/dL (95% CI [1.64–4.12]) and 2.90 mg/dL (95% CI [1.71–4.09])), HDL-C significantly increased (0.97 mg/dL (95% CI [0.45–1.48]) and 2.39 mg/dL (1.89–2.88)), and TG significantly decreased (3.12 mg/dL (95% CI [0.49–5.75]) and 6.18 mg/dL (95% CI [3.65–8.72])). Overall, the table suggests that coffee consumption is associated with changes in lipid profiles in Taiwanese men and women. Additionally, the analysis has been adjusted for various demographic and lifestyle factors to minimize confounding effects.

The adjusted serum lipid profile changes based on coffee consumption in postmenopausal and premenopausal women are shown in Table 3. For the postmenopausal women, compared with non-drinkers, all of the lipid profiles had significant changes in the group that consumed <1 cup/day and ≥1 cup/day, except for the group that consumed <1 cup/day regarding TG. Compared with non-drinkers, the group that consumed <1 cup/day and ≥1 cup/day showed the following: TC significantly increased (3.84 mg/dL (95% CI [2.05–5.63]) and 4.80 mg/dL (95% CI [2.95–6.65])); LDL-C significantly increased (3.03 mg/dL (95% CI [1.43–4.64]) and 2.46 mg/dL (95% CI [0.80–4.11])); and HDL significantly increased (0.92 mg/dL (95% CI [0.26–1.57]) and 2.87 mg/dL (95% CI [2.20–3.54])). Moreover, TG significantly decreased (6.47 mg/dL (95% CI [3.05–9.88])) in the group that consumed ≥1 cup/day. For the premenopausal women, compared with non-drinkers, all of the lipid profiles had significant changes in the group that consumed ≥1 cup/day, but only HDL-C and TG had significant changes in the group that consumed <1 cup/day. Compared with non-drinkers, the group that consumed ≥1 cup/day showed the following: TC significantly increased (3.45 mg/dL (95% CI [1.57–5.34])); LDL-C significantly increased (2.46 mg/dL (95% [0.79–4.13])); HDL-C significantly increased (1.72 mg/dL (95% CI [1.00–2.45])); and TG significantly decreased (7.38 mg/dL (95% CI [3.58–11.19])). Compared with non-drinkers, the group that consumed <1 cup/day showed the following: HDL-C significantly increased (1.00 mg/dL (95% CI [0.17–1.83])); and TG significantly decreased (6.85 mg/dL (95% CI [2.50–11.20])).

## 4. Discussion

Our study demonstrated that habitual coffee consumption caused different changes in serum lipid profiles between Taiwanese men and women. Although serum TC and LDL-C levels significantly increased in men and women based on whether they were habitual coffee consumers, premenopausal women only demonstrated an increase in serum TC and LDL-C levels in the group that consumed ≥1 cup/day. Men and postmenopausal women were more sensitive to the effects of habitual drinking on serum TC and LDL-C levels than premenopausal women. On the other hand, compared with non-drinkers, women had a significant increase in HDL-C serum levels in the group with habitual coffee consumption, and the same results were observed for premenopausal and postmenopausal women. However, men only exhibited a significant change when they consumed ≥1 cup/day. The effect of habitual coffee consumption on serum HDL-C levels exhibited sex differences, but there was no difference among women based on their menopausal status. Compared with non-drinkers, the serum TG levels significantly decreased in women with habitual coffee consumption, but only decreased in the group that consumed ≥1 cup/day in men. Moreover, postmenopausal women only exhibited this decrease in the group that consumed ≥1 cup/day. The effect of habitual coffee consumption on serum TG levels may change with menopausal status in women. In summary, premenopausal women seemed less sensitive to the effect of coffee drinking on raising serum TC and LDL-C levels and more sensitive to the effect of coffee drinking on decreasing serum TG levels than men and postmenopausal women. The effect of coffee consumption on serum HDL-C levels exhibited sex differences; however, there seemed to be no difference between the groups of women based on their menopausal status.

Coffee contains several bioactive compounds, including caffeine, chlorogenic acids, trigonelline, and diterpenes, which are involved in lipid metabolism [33]. Diterpenes are regarded as cholesterol-increasing compounds, including cafestol and kahweol [34]. The concentration of diterpenes depends on the preparation approach [8] and coffee species [2]. According to in vivo studies, caffeine, chlorogenic acids, and trigonelline were reported to be involved in lipid metabolism and may potentially influence serum TC, LDL-C, and TG levels [35,36,37,38,39]. In addition, the influence of sex differences on lipid metabolism in habitual coffee consumers may change the results of serum lipid profiles, and some studies have reported that the potential reasons are genetic variability [16] and serum estradiol level changes [14,40]. These possible factors, such as the ratio of bioactive compounds from each type of coffee, study design, and participants’ characteristics, can cause inconsistent changes in serum lipid profiles.

The coffee drinking habits in Taiwan have gradually developed over the past two decades, and the flavor of the coffee types and daily intake volume for Taiwanese people are also unique [13]. Our study used data from the TWB database to discuss the relationship between different coffee intake volumes and serum lipid profiles among men and women, as well as premenopausal and postmenopausal women.

Some studies that enrolled short-term clinical trials ranging from 4 weeks to 6 months in small-size populations revealed that coffee consumption increased serum TC and LDL-C levels [3,41,42,43,44]. Moreover, two nationwide population studies from the UK Biobank demonstrated that habitual coffee intake increased serum TC and LDL-C levels even in the group that consumed <1 cup/day [45,46]. This is consistent with our study results, which revealed that both men and women had increased serum TC and LDL-C levels based on habitual coffee consumption. Nevertheless, the finding that only drinking ≥1 cup of coffee per day could change the serum TC and LDL-C levels in premenopausal women implies that premenopausal women are not as sensitive as men and postmenopausal women to the effect of coffee consumption. During their fertile period, women have higher serum estradiol levels, which is synthesized by LDL-C in the ovary; hence, when they reach menopause, a decrease in estradiol synthesis due to menopause means that LDL-C is no longer used for synthesizing estradiol and remains in the systemic circulation [14]. In addition, a study assessed the relationship between caffeine and caffeinated beverage intake and reproductive hormones in healthy premenopausal women. It disclosed that caffeine intake ≥200 mg/day was positively associated with free estradiol concentrations among Asian women (β = 0.61; 95% CI: 0.31–0.92) [40], which might cause potential protective effects on serum LDL-C levels rising due to diterpenes intake from coffee consumption in premenopausal women. Caffeine and coffee intake were both associated with profiles of estrogen metabolism in premenopausal women [17,47]. The decreased concentration of serum estradiol after menopause and the amount of caffeine intake may play important roles in the difference in sensitivity of serum TC and LDL-C levels to the habitual coffee intake volumes. 

In this study, habitual coffee consumption increased serum HDL-C levels in both men and women. Meanwhile, compared to men, women were more sensitive to the effects of coffee drinking, regardless of their menopausal status. Two meta-analyses that enrolled a short-term clinical trial with a small sample size revealed that only three studies demonstrated that habitual coffee consumption increased serum HDL levels, and other studies had no significant findings [3,4]. A nationwide population study from the UK Biobank revealed that the daily coffee consumption of ≥1 cup per day increased serum HDL-C levels, as did ground coffee and instant coffee [45]. Another UK Biobank study revealed that coffee consumption was significantly associated with higher HDL-C and apoA-1 concentrations; however, this was only observed for low to medium consumption and did not follow a dose–response pattern [46]. Furthermore, some articles have discussed sex differences in serum HDL-C levels after coffee consumption [48,49]. One Korean study indicated that the potential effects of coffee intake on dyslipidemia risk depend on genetic variants in the ADORA gene family in a sex-specific manner. Moreover, genetic heterogeneity may also imply inconsistent results in the effects of habitual coffee consumption on serum lipid levels among studies with regional and racial differences. 

Our study revealed that coffee consumption had a favorable effect on serum TG levels in both men and women. The premenopausal women that were habitual coffee consumers exhibited lower serum TG levels compared with non-drinkers, but men and postmenopausal women only exhibited this when they consumed ≥1 cup/day. Many short-term and small-sample-size clinical trials from several meta-analyses revealed high heterogeneity among these studies, and most showed an insignificant relationship between habitual coffee consumption and serum TG levels; only a few studies showed increased serum TG levels [3,4]. However, nationwide population-based cohort studies in the UK revealed that medium- to high-level coffee consumption decreased serum TG levels [45,46]. Two cross-sectional cohort studies also revealed that habitual coffee consumption decreased the prevalence of hypertriglyceridemia [50,51]. Hence, the inconsistent results from those studies may have been due to differences in the trial duration, sample size, and genetic variants [16]. In addition, our study demonstrated that in premenopausal women, habitual coffee consumption reduced serum TG levels more easily than in men and postmenopausal women, implying that serum estradiol plays an important role in serum TG regulation.

Despite efforts to balance the confounding factors, this study had several limitations. First, despite the large database, our cross-sectional study design precluded the confirmation of causal associations. Second, many variables were self-reported, which may have caused an overestimation or underestimation of the prevalence of risk factors. However, our study participants were from the follow-up population, which was more reliable for information collection than those who underwent the investigation only once. Third, although we adjusted for a broad variety of well-known potential confounders related to socioeconomic status and lifestyle, there could be confounding factors such as diet or the amount of milk or sugar added to the coffee, which were not accounted for in the analysis. Finally, our study only enrolled middle-aged to older adult participants; therefore, findings from younger populations could not be included in our results.

## 5. Conclusions

Our study demonstrated that habitual coffee consumption altered the serum lipid profiles of Taiwanese men and women. Compared with premenopausal women, both men and postmenopausal women are more likely to exhibit increased serum TC and LDL-C levels, but not decreased serum TG levels. Moreover, menopausal status may play an important role in modulating the effect of habitual coffee intake on dyslipidemia, and premenopausal women may potentially receive more benefits from habitual coffee consumption than men and postmenopausal women. Further study about the influence of coffee consumption on lipid profiles is needed, and our study emphasized the importance of individual discussion in populations with different sexual and menopausal status.

## Figures and Tables

**Table 1:** Lipid profiles and baseline characteristics based on different coffee consumption volumes in Taiwanese men and women aged 30–79 years (2008–2020).

Number (%) (N = 23,628)	Male N = 4887 (59.5%)	N = 1558 (19.0%)	N = 1763 (21.5%)		Female N = 8971 (58.2%)	N = 3003 (19.5%)	N = 3446 (22.3%)	
	NCD	<1 Cup/Day	≥1 Cup/Day	*p*-Value	NCD	<1 Cup/Day	≥1 Cup/Day	*p*-Value
Lipid Profile (mg/dL)								
Total Cholesterol	188.05 (34.85)	190.88 (34.25)	193.52 (34.36)	<0.001 *	197.97 (35.20)	201.59 (34.63)	200.98 (34.35)	<0.001 *
LDL-C	117.75 (31.04)	120.65 (30.49)	123.55 (31.22)	<0.001 *	119.99 (30.90)	123.00 (30.67)	121.98 (30.57)	<0.001 *
HDL-C	48.11 (11.44)	48.69 (11.30)	48.86 (11.07)	0.031 *	57.73 (13.27)	58.69 (13.31)	59.87 (13.21)	<0.001 *
Triglycerides	133.02 (110.96)	130.95 (95.30)	128.16 (88.87)	0.232	107.07 (70.73)	104.03 (63.00)	99.75 (59.88)	<0.001 *
Age (Years)	55.13 (11.04)	54.65 (10.79)	53.12 (10.71)	<0.001	54.81 (10.18)	54.34 (9.72)	51.80 (9.45)	<0.001 *
Age								
30–50 years								
50–60 years								
60–80 years								
Age ≥ 65 years	1192 (24.4)	327 (21.0)	317 (18.0)	<0.001	1679 (18.7)	476 (15.9)	347 (10.1)	<0.001 *
BMI (Kg/m^2^)	24.99 (3.42)	25.20 (3.22)	25.28 (3.28)	0.002 *	23.58 (3.71)	23.74 (3.42)	23.86 (3.59)	<0.001 *
BMI				0.005 *				0.004 *
BMI < 24 kg/m^2^	1982 (40.6)	583 (37.4)	632 (35.8)		5477 (61.1)	1763 (58.7)	2000 (58.0)	
BMI 24–27 kg/m^2^	1733 (35.5)	600 (38.5)	677 (38.4)		2081 (23.2)	767 (25.5)	842 (24.4)	
BMI > 27 kg/m^2^	1172 (24.0)	375 (24.1)	454 (25.8)		1413 (15.8)	473 (15.8)	604 (17.5)	
Education Level (College or Above)	2770 (56.7)	1038 (66.6)	1174 (66.6)	<0.001 *	3681 (41.0)	1398 (46.6)	1738 (50.4)	<0.001 *
Marriage Status (Married)	4187 (85.7)	1390 (89.2)	1501 (85.1)	0.001 *	6622 (73.8)	2216 (73.8)	2507 (72.8)	0.462
Occupational Status (Employed)	3166 (64.8)	1054 (67.7)	1275 (72.3)	<0.001 *	4647 (51.8)	1663 (55.4)	2155 (62.5)	<0.001 *
Place of Residence				<0.001 *				0.715
Urban area	2581 (52.8)	842 (54.0)	1040 (59.0)		5092 (56.8)	1727 (57.5)	1991 (57.8)	
Suburban area	1933 (39.6)	586 (37.6)	619 (35.1)		3337 (37.2)	1085 (36.1)	1244 (36.1)	
Rural area	373 (7.6)	130 (8.3)	104 (5.9)		542 (6.0)	191 (6.4)	211 (6.1)	
Monthly Personal Income				<0.001 *				<0.001 *
0–30,000 NTD	1181 (24.2)	268 (17.2)	306 (17.4)		4369 (48.7)	1291 (43.0)	1382 (40.1)	
30,001–60,000 NTD	1767 (36.2)	585 (37.5)	683 (38.7)		2377 (26.5)	904 (30.1)	1134 (32.9)	
60,001 NTD or more	1939 (39.7)	705 (45.3)	774 (43.9)		2225 (24.8)	808 (26.9)	930 (27.0)	
Cigarette Smoking Status				<0.001 *				<0.001 *
Non-smokers	2825 (57.8)	842 (54.0)	848 (48.1)		8705 (97.0)	2881 (95.9)	3219 (93.4)	
Former smokers	1182 (24.2)	459 (29.5)	496 (28.1)		135 (1.5)	68 (2.3)	108 (3.1)	
Current smokers	880 (18.0)	257 (16.5)	419 (23.8)		131 (1.5)	54 (1.8)	119 (3.5)	
Alcohol Drinking Status				<0.001 *				<0.001 *
Non-drinkers	3800 (77.8)	1171 (75.2)	1300 (73.7)		8766 (97.7)	2896 (96.4)	3271 (94.9)	
Former drinkers	383 (7.8)	104 (6.7)	155 (8.8)		82 (0.9)	32 (1.1)	41 (1.2)	
Current Drinkers	704 (14.4)	283 (18.2)	308 (17.5)		123 (1.4)	75 (2.5)	134 (3.9)	
Habitual Tea Drinking	1537 (31.5)	557 (35.8)	563 (31.9)	0.006 *	1414 (15.8)	693 (23.1)	826 (24.0)	<0.001 *
Vegetarian	196 (4.0)	59 (3.8)	59 (3.3)	0.458	606 (6.8)	138 (4.6)	156 (4.5)	<0.001 *
Regular Exercise	2305 (47.2)	811 (52.1)	872 (49.5)	0.002 *	4255 (47.4)	1470 (49.0)	1483 (43.0)	<0.001 *
Daily Sleeping Hours (Hour)	6.74 (1.10)	6.81 (0.97)	6.74 (1.00)	0.068	6.68 (1.14)	6.73 (1.08)	6.81 (1.07)	<0.001 *
Menopause					5924 (66.0)	1905 (63.4)	1800 (52.2)	<0.001 *
Exogenous Hormones					1396 (15.6)	450 (15.0)	439 (12.7)	<0.001 *

* *p*-value < 0.05. Values are means with SD in parentheses (continuous variables) or percentage (categorical variables). NCD, non-coffee drinker; LDL-C, low-density lipoprotein cholesterol; HDL-C, high-density lipoprotein cholesterol.

**Table 2:** General linear model coefficients adjusted by the basic characteristics of the association between lipid profiles and coffee consumption in Taiwanese men and women.

Index	Males			Females		
	NCD	<1 Cup/Day	≥1 Cup/Day	NCD	<1 Cup/Day	≥1 Cup/Day
Number	4875	1639	1821	8547	2872	3295
TC						
*β*-coefficient [95% CI] mg/dL	Ref.	2.41 (0.45–4.38)	4.52 (2.63–6.40)	Ref.	3.53 (2.14–4.93)	4.83 (3.49–6.17)
*p*-value		0.016 *	<0.001 *		<0.001 *	<0.001 *
LDL-C						
*β*-coefficient [95% CI] mg/dL	Ref.	2.75 (0.99–4.51)	5.10 (3.42–6.79)	Ref.	2.88 (1.64–4.12)	2.90 (1.71–4.09)
*p*-value		0.002 *	<0.001 *		<0.001 *	<0.001 *
HDL-C						
*β*-coefficient [95% CI] mg/dL	Ref.	0.53 (−0.07–1.13)	0.95 (0.37–1.53)	Ref.	0.97 (0.45–1.48)	2.39 (1.89–2.88)
*p*-value		0.085	0.001 *		<0.001 *	<0.001 *
TG						
*β*-coefficient [95% CI] mg/dL	Ref.	2.90 (−9.40–1.96)	−9.62 (−15.07–4.17)	Ref.	−3.12 (−5.75–0.49)	−6.18 (−8.72–3.65)
*p*-value		0.200	0.001 *		0.020 *	<0.001 *

Ref., reference; * *p*-value < 0.05; *β*, *β*-coefficient; CI, confidence interval; NCD, non-coffee drinkers; LDL-C, low-density lipoprotein cholesterol; HDL-C, high-density lipoprotein cholesterol; TC, total cholesterol; TG, triglycerides. Adjusted *β*-coefficient: reference = non-coffee consumer, adjusted for age, body mass index, education level, marital status, occupational status, place of residence, cigarette smoking status, secondhand smoke exposure, alcohol consumption, habitual tea consumption, regular exercise, vegetarian status, daily sleep hours, monthly income, and menopause (for females).

**Table 3:** General linear model coefficients adjusted by the basic characteristics of the association between lipid profiles and coffee consumption in postmenopausal and premenopausal women.

Index	Postmenopausal Women			Premenopausal Women		
	NCD	<1 Cup/Day	≥1 Cup/Day	NCD	<1 Cup/Day	≥1 Cup/Day
Number	5742	1867	1776	2805	1005	1519
TC						
*β*-coefficient [95% CI] mg/dL	Ref.	3.84 (2.05–5.63)	4.80 (2.95–6.65)	Ref.	1.95 (−0.21–4.11)	3.45 (1.57–5.34)
*p*-value		<0.001 *	<0.001 *		0.076	<0.001 *
LDL-C						
*β*-coefficient [95% CI] mg/dL		3.03 (1.43–4.64)	2.46 (0.80–4.11)	Ref.	1.81 (−0.10–372)	2.46 (0.79–4.13)
*p*-value		<0.001 *	0.004 *		0.063	0.004 *
HDL-C						
*β*-coefficient [95% CI] mg/dL	Ref.	0.92 (0.26–1.57)	2.87 (2.20–3.54)	Ref.	1.00 (0.17–1.83)	1.72 (1.00–2.45)
*p*-value		0.006 *	<0.001 *		0.018 *	<0.001 *
TG						
*β*-coefficient [95% CI] mg/dL	Ref.	−1.38 (−4.68–1.93)	−6.47 (−9.88–3.05)	Ref.	−6.85 (−11.20–2.50)	−7.38 (−11.19–3.58)
*p*-value		0.414	<0.001 *		0.002 *	<0.001 *

Ref., reference; * *p*-value < 0.05; β, β-coefficient; CI, confidence interval; NCD, non-coffee drinkers; LDL-C, low-density lipoprotein cholesterol; HDL-C, high-density lipoprotein cholesterol; TC, total cholesterol; TG, triglycerides. Adjusted-coefficient: reference = non-coffee consumer, adjusted for age, body mass index, education level, marital status, occupational status, place of residence, monthly income, cigarette smoking status, alcohol consumption, habitual tea consumption, regular exercise, vegetarian status, daily sleeping hours, menopause, and hormone supplementation (for females).

## Data Availability

Not applicable.
